# (*Z*)-1,1-Dicyano-2-(4-fluoro­phen­yl)-3-(1-hexyl­pyridin-1-ium-4-yl)prop-2-en-1-ide

**DOI:** 10.1107/S160053681105166X

**Published:** 2011-12-10

**Authors:** Wen-Hui Hao, Cheng Wang, Gang Qian, Zhi-Yuan Wang

**Affiliations:** a Department of Chemistry, Carleton University, Ottawa, Ontario, Canada K1S 5B6; b Key Laboratory of Functional Materials and Key Laboratory of Polymer Functional Materials, Heilongjiang University, Harbin 150080, People’s Republic of China

## Abstract

The title compound, C_22_H_22_FN_3_, exists as a zwitterion with the negative charge on the dicyano­methanide group and the positive charge on the pyridinium N atom. The mol­ecule adopts a *Z* conformation about the central C=C bond. The dihedral angle between the pyridinium and benzene rings is 65.65 (5)°. Weak C—H⋯N hydrogen bonding is present in the crystal structure.

## Related literature

For details of zwitterionic chromophores and their applications, see: Hao (2011 ▶); Hao *et al.* (2011 ▶). For related structures, see: Metzger & Heimer (1984 ▶); Bell *et al.* (2002 ▶); Cole *et al.* (1997 ▶); Szablewski *et al.* (1997 ▶); Xiong *et al.*(2008 ▶). For the synthesis, see: Hao (2011 ▶). For standard bond lengths, see: Allen *et al.* (1987 ▶). 
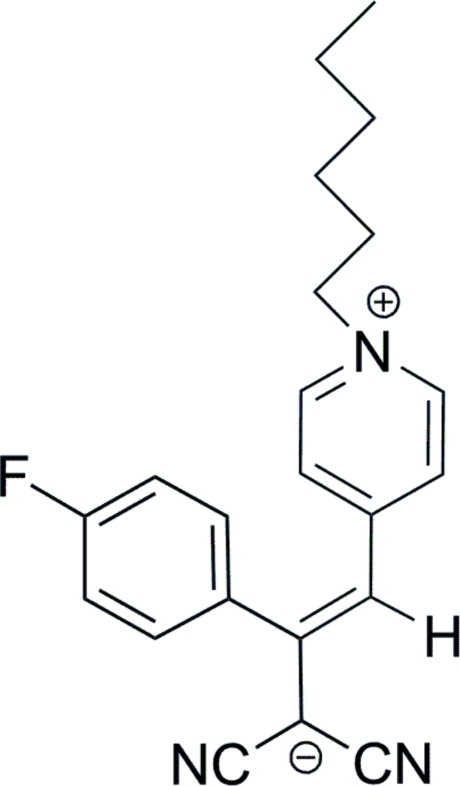


## Experimental

### 

#### Crystal data

C_22_H_22_FN_3_
*M* *_r_* = 347.43
Monoclinic, 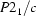

*a* = 10.485 (3) Å
*b* = 8.809 (2) Å
*c* = 21.313 (5) Å
β = 100.628 (4)°
*V* = 1934.7 (8) Å^3^
*Z* = 4
Mo *K*α radiation
μ = 0.08 mm^−1^
*T* = 295 K
0.24 × 0.21 × 0.18 mm

#### Data collection

Rigaku R-AXIS RAPID diffractometer
Absorption correction: multi-scan (*ABSCOR*; Higashi, 1995 ▶) *T*_min_ = 0.982, *T*_max_ = 0.986
9762 measured reflections
3406 independent reflections
2154 reflections with *I* > 2σ(*I*)
*R*_int_ = 0.046

#### Refinement

*R*[*F* ^2^ > 2σ(*F* ^2^)] = 0.043
*wR*(*F* ^2^) = 0.080
*S* = 1.01
3406 reflections
237 parameters
H-atom parameters constrained
Δρ_max_ = 0.13 e Å^−3^
Δρ_min_ = −0.14 e Å^−3^

### 

Data collection: *RAPID-AUTO* (Rigaku, 1998 ▶); cell refinement: *RAPID-AUTO*; data reduction: *CrystalStructure* (Rigaku/MSC, 2002 ▶); program(s) used to solve structure: *SHELXTL* (Sheldrick, 2008 ▶); program(s) used to refine structure: *SHELXTL*; molecular graphics: *SHELXTL*; software used to prepare material for publication: *SHELXTL*.

## Supplementary Material

Crystal structure: contains datablock(s) I, global. DOI: 10.1107/S160053681105166X/xu5392sup1.cif

Structure factors: contains datablock(s) I. DOI: 10.1107/S160053681105166X/xu5392Isup2.hkl

Supplementary material file. DOI: 10.1107/S160053681105166X/xu5392Isup3.cdx

Supplementary material file. DOI: 10.1107/S160053681105166X/xu5392Isup4.cml

Additional supplementary materials:  crystallographic information; 3D view; checkCIF report

## Figures and Tables

**Table 1:** Hydrogen-bond geometry (Å, °)

*D*—H⋯*A*	*D*—H	H⋯*A*	*D*⋯*A*	*D*—H⋯*A*
C3—H3*A*⋯N2^i^	0.93	2.61	3.535 (3)	175
C16—H16*A*⋯N1^ii^	0.93	2.51	3.354 (3)	151

Symmetry codes: (i) 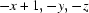; (ii) 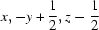.

**Fig. 1.**
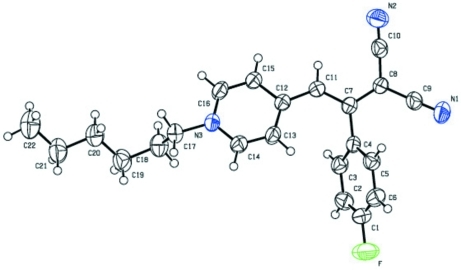
The Molecular structure of compound (I) with displacement ellipsoids drawn at the 30% probability level. Hydrogen atoms were omitted for clarity.

**Fig. 2.**
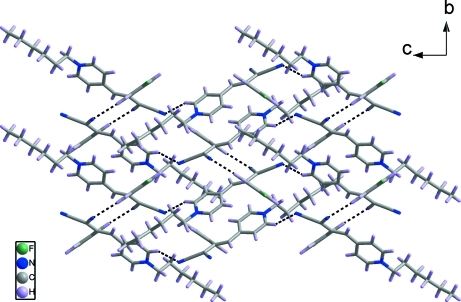
Partial packing view showing the 2-D layer structure of compound (I) through two types of weak intermolecular C–H..N hydrogen bonds. Hydrogen bonds are shown by dashed lines.

**Fig. 3.**
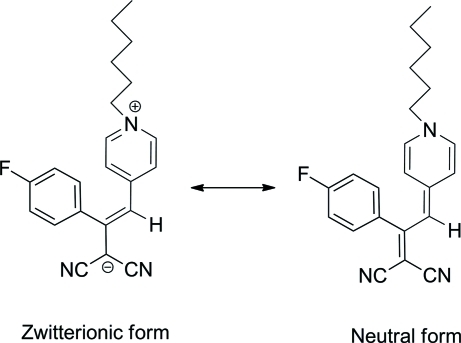
Resonance structures of the title compound.

## supplementary crystallographic information

### Comment

Structure determination of the title compound (I), C_22_H_22_FN_3_ (Fig. 1), was performed as a part of a project in our laboratory on the synthesis of new series of D^+^-π-A^-^ zwitterionic chromophores for electro-optic and near infrared chemosensor applications (Hao, 2011; Hao *et al.*, 2011).

The title compound (I) crystallizes as a zwitterion in which the negative charge on the dicyanomethanide (–C(CN)_2_) group and the positive charge on the N atom in the pyridinium ring. It presents a Z configuration of the central C7═C11 double bond with the torsion angle C12—C11—C7—C8 being -168.25 (17) °.

The bond length between C7 and C11 (1.3837 (19) Å) is similar to the value observed in other zwitterionic compounds (Metzger & Heimer, 1984; Bell *et al.*, 2002), which clearly indicates the double bond π-bridge separating the pyridinium and dicyanomethanide groups. A significant displacement of electron density (or charge transfer)from the pyridinium ring (donor) to the –C(CN)_2_ group (acceptor) was confirmed, corresponding to a large contribution of the zwitterionic resonance structure in compound (I).

The bond lengths between C7 and C8 (1.4078 (20) Å), C7 and C11 (1.3837 (19) Å) suggest electron delocalization among the three carbon atoms. However, more single bond characteristic is observed between C7 and C8;also evident from the observed two C—CN bonds (1.4111 (22) Å and 1.4201 (23) Å) in compound (I) *versus* 1.427 Å in typical 7,7,8,8-tetracyanoquino-dimethanes(TCNQs), indicating substantial negative charge localization within the –C(CN)_2_ group, and elongated CN (1.1514 (20) Å and 1.1472 (20) Å) bond compared to that in typical TCNQs (1.144 Å) (Allen *et al.*, 1987) indicating a result of charge resonant stabilization *via* the two CN groups.

Although the bond lengths in the conjugated bridge and acceptor part clearly demonstrated a zwitterionic molecular structure of compound (I), the bond length of the pyridinium ring is quinoidal rather than aromatic. The C13—C14 and C15—C16 bonds (with bond lengths of 1.3516 (20) Å, 1.3491 (21) Å) are shorter than the C12—C13 and C12—C15 bonds (1.4088 (21) Å and 1.4125 (20) Å). Similar phenomena have also been reported for several TCNQ and 7,8-di(alkoxycarbonyl)-7,8- dicyanoquinodimethane zwitterionic adducts (Cole *et al.*, 1997; Szablewski *et al.*, 1997; Xiong *et al.*, 2008). Therefore, the best description of the ground state structure of compound (I) is the combination of the two limiting forms (Fig. 3), zwitterionic and neutral forms with predominantly zwitterionic structure.

As shown in Fig.2, two types of weak intermolecular C–H..N hydrogen bonds connect adjacent molecules, forming a 2-D layer structure in the *bc* plane with the bond lengths and angles being 3.354 (3) Å, 150.77 (1) ° (C16—H16A···N1) and 3.535 (2) Å, 174.79 (1) ° (C3—H3A···N2).

### Experimental

Under anhydrous and oxygen-free conditions, to a 50 ml round-bottomed flask malononitrile (0.50 g, 7.4 mmol), sodium hydride (0.30 g, 60%, 7.5 mmol) and 2-(4-fluorophenyl)-3-pyridine-4-yl-acrylonitrile bromide salt (Hao, 2011) (1.0 g, 2.6 mmol) in 20 ml of THF were added at 0°C. After 30 min, the insoluble inorganic salt was removed by filtration, and the filtrate solution was concentrated under reduced pressure. The residue was purified by column chromatography (flash, mixture of acetone and hexane with the ratio 1:1) to produce (I) (0.56 g, 63% yield). Orange-red crystals were obtained from a hexane/acetone solution of (I) by slow evaporation at room temperature.

### Refinement

The H atoms attached to the carbon atoms were placed in calculated positions, with C—H = 0.93–0.97 Å, and were refined in the riding-model approximation with *U*_iso_(H) = 1.5*U*_eq_(C) for methyl H atoms and 1.2*U*_eq_(C) for the others,

### Crystal data

**Table:**

C_22_H_22_FN_3_	*F*(000) = 736
*M**_r_* = 347.43	*D*_x_ = 1.193 Mg m^−^^3^
Monoclinic, *P*2_1_/*c*	Mo *K*α radiation, λ = 0.71073 Å
Hall symbol: -P 2ybc	Cell parameters from 7543 reflections
*a* = 10.485 (3) Å	θ = 2.0–25.0°
*b* = 8.809 (2) Å	µ = 0.08 mm^−^^1^
*c* = 21.313 (5) Å	*T* = 295 K
β = 100.628 (4)°	Prism, red
*V* = 1934.7 (8) Å^3^	0.24 × 0.21 × 0.18 mm
*Z* = 4	

### Data collection

**Table:**

Rigaku R-AXIS RAPID diffractometer	3406 independent reflections
Radiation source: fine-focus sealed tube	2154 reflections with *I* > 2σ(*I*)
graphite	*R*_int_ = 0.046
Detector resolution: 10.000 pixels mm^-1^	θ_max_ = 25.0°, θ_min_ = 1.9°
ω scans	*h* = −12→9
Absorption correction: multi-scan (*ABSCOR*; Higashi, 1995)	*k* = −10→10
*T*_min_ = 0.982, *T*_max_ = 0.986	*l* = −24→25
9762 measured reflections	

### Refinement

**Table:**

Refinement on *F*^2^	Primary atom site location: structure-invariant direct methods
Least-squares matrix: full	Secondary atom site location: difference Fourier map
*R*[*F*^2^ > 2σ(*F*^2^)] = 0.043	Hydrogen site location: inferred from neighbouring sites
*wR*(*F*^2^) = 0.080	H-atom parameters constrained
*S* = 1.01	*w* = 1/[σ^2^(*F*_o_^2^) + (0.0208*P*)^2^] where *P* = (*F*_o_^2^ + 2*F*_c_^2^)/3
3406 reflections	(Δ/σ)_max_ < 0.001
237 parameters	Δρ_max_ = 0.13 e Å^−^^3^
0 restraints	Δρ_min_ = −0.14 e Å^−^^3^

### Special details

**Table:**

Experimental. Melting point: 167 °C; UV-Vis: λ_max_ = 486 nm (DMF); FTIR (KBr, cm^-1^): 2192, 1640, 1571, 1450; ^1^H NMR (400 MHz, (CD_3_)_2_SO): (δ p.p.m.): 7.81 (2*H*, d, J = 7.1 Hz), 7.36 (2*H*, d, J = 8.7 Hz), 7.32 (2*H*, d, J = 8.7 Hz), 6.34 (2*H*, d, J = 7.1 Hz), 5.88 (1*H*, s), 3.92 (2*H*, t), 1.22 (6*H*, m), 0.83 (3*H*, t); ^13^ C NMR (100 MHz, (CD_3_)_2_SO: (δ p.p.m.): 161.2, 151.4, 139.8, 133.7, 130.0, 129.9, 119.8, 119.2, 118.2, 116.6, 116.4, 101.7, 56.7, 30.4,29.8, 24.9, 21.8, 13.7; TOF HRMS (ESI, DMF/Acetonitrile 1:1, m/*z*): Calculated value: 347.1798 [*M*]^+^. Found: 348.1776 [*M* + H]^+^, 370.1608 [*M* + Na]^+^.

### Fractional atomic coordinates and isotropic or equivalent isotropic displacement parameters (Å^2^)

**Table:**

	*x*	*y*	*z*	*U*_iso_*/*U*_eq_	
F	0.00950 (11)	0.32278 (14)	0.14713 (6)	0.0895 (4)	
N1	0.52466 (16)	0.0019 (2)	0.21926 (7)	0.0776 (6)	
N2	0.79360 (16)	0.0489 (2)	0.08676 (7)	0.0706 (5)	
N3	0.31701 (13)	0.49453 (16)	−0.14317 (6)	0.0446 (4)	
C1	0.12274 (18)	0.2874 (2)	0.12778 (9)	0.0548 (5)	
C2	0.11792 (17)	0.1996 (2)	0.07511 (9)	0.0544 (5)	
H2A	0.0392	0.1629	0.0530	0.065*	
C3	0.23295 (17)	0.16598 (19)	0.05513 (8)	0.0472 (5)	
H3A	0.2314	0.1067	0.0189	0.057*	
C4	0.35090 (15)	0.21967 (18)	0.08855 (7)	0.0384 (4)	
C5	0.34999 (16)	0.30647 (19)	0.14272 (8)	0.0457 (5)	
H5A	0.4282	0.3411	0.1662	0.055*	
C6	0.23523 (19)	0.3429 (2)	0.16267 (8)	0.0546 (5)	
H6A	0.2349	0.4029	0.1985	0.065*	
C7	0.47440 (15)	0.17940 (18)	0.06776 (7)	0.0384 (4)	
C8	0.56746 (16)	0.10162 (19)	0.11230 (7)	0.0416 (4)	
C9	0.54269 (17)	0.0483 (2)	0.17131 (9)	0.0507 (5)	
C10	0.6924 (2)	0.0696 (2)	0.09897 (8)	0.0483 (5)	
C11	0.49704 (15)	0.21053 (18)	0.00714 (7)	0.0429 (4)	
H11A	0.5680	0.1601	−0.0035	0.051*	
C12	0.43029 (15)	0.30640 (18)	−0.04143 (7)	0.0394 (4)	
C13	0.33424 (16)	0.4152 (2)	−0.03586 (8)	0.0474 (5)	
H13A	0.3067	0.4261	0.0030	0.057*	
C14	0.28129 (16)	0.5040 (2)	−0.08563 (8)	0.0487 (5)	
H14A	0.2180	0.5740	−0.0800	0.058*	
C15	0.46572 (16)	0.30234 (19)	−0.10225 (8)	0.0474 (5)	
H15A	0.5303	0.2353	−0.1091	0.057*	
C16	0.40928 (17)	0.3923 (2)	−0.15072 (8)	0.0503 (5)	
H16A	0.4343	0.3838	−0.1902	0.060*	
C17	0.25548 (16)	0.5900 (2)	−0.19749 (8)	0.0493 (5)	
H17A	0.3214	0.6252	−0.2206	0.059*	
H17B	0.2167	0.6784	−0.1814	0.059*	
C18	0.15272 (17)	0.5044 (2)	−0.24250 (8)	0.0600 (5)	
H18A	0.0928	0.4587	−0.2183	0.072*	
H18B	0.1935	0.4231	−0.2623	0.072*	
C19	0.07691 (18)	0.6055 (2)	−0.29453 (8)	0.0680 (6)	
H19A	0.0074	0.5459	−0.3192	0.082*	
H19B	0.0372	0.6869	−0.2743	0.082*	
C20	0.15573 (18)	0.6746 (2)	−0.33983 (8)	0.0630 (6)	
H20A	0.2031	0.5948	−0.3569	0.076*	
H20B	0.2187	0.7443	−0.3164	0.076*	
C21	0.07270 (19)	0.7591 (3)	−0.39474 (9)	0.0756 (6)	
H21A	0.0056	0.6915	−0.4162	0.091*	
H21B	0.0303	0.8436	−0.3778	0.091*	
C22	0.1496 (2)	0.8187 (2)	−0.44271 (9)	0.0883 (7)	
H22A	0.2180	0.8831	−0.4215	0.132*	
H22B	0.0935	0.8757	−0.4750	0.132*	
H22C	0.1863	0.7350	−0.4622	0.132*	

### Atomic displacement parameters (Å^2^)

**Table:**

	*U*^11^	*U*^22^	*U*^33^	*U*^12^	*U*^13^	*U*^23^
F	0.0586 (8)	0.1145 (11)	0.1030 (10)	0.0154 (7)	0.0347 (7)	−0.0070 (8)
N1	0.0966 (14)	0.0953 (15)	0.0443 (11)	0.0314 (10)	0.0222 (9)	0.0153 (10)
N2	0.0533 (12)	0.0938 (14)	0.0638 (12)	0.0117 (10)	0.0080 (9)	−0.0081 (10)
N3	0.0499 (10)	0.0481 (9)	0.0359 (9)	0.0020 (7)	0.0080 (7)	0.0045 (7)
C1	0.0471 (13)	0.0640 (14)	0.0584 (13)	0.0105 (10)	0.0230 (10)	0.0063 (11)
C2	0.0425 (12)	0.0657 (14)	0.0529 (13)	−0.0029 (10)	0.0036 (9)	0.0041 (11)
C3	0.0491 (12)	0.0540 (12)	0.0378 (10)	−0.0025 (9)	0.0058 (9)	−0.0035 (9)
C4	0.0431 (11)	0.0412 (11)	0.0308 (10)	0.0009 (8)	0.0064 (8)	0.0034 (8)
C5	0.0461 (12)	0.0481 (12)	0.0419 (11)	0.0000 (9)	0.0050 (9)	−0.0025 (9)
C6	0.0626 (14)	0.0568 (13)	0.0466 (12)	0.0067 (10)	0.0162 (10)	−0.0075 (10)
C7	0.0427 (11)	0.0378 (10)	0.0345 (10)	−0.0032 (8)	0.0069 (8)	−0.0035 (8)
C8	0.0423 (12)	0.0500 (12)	0.0327 (10)	0.0035 (8)	0.0078 (8)	−0.0015 (9)
C9	0.0554 (13)	0.0590 (13)	0.0364 (11)	0.0131 (9)	0.0051 (9)	−0.0009 (10)
C10	0.0531 (13)	0.0527 (12)	0.0360 (11)	0.0030 (10)	0.0001 (9)	−0.0029 (9)
C11	0.0445 (11)	0.0463 (11)	0.0389 (10)	0.0054 (8)	0.0104 (8)	0.0001 (9)
C12	0.0408 (10)	0.0416 (11)	0.0360 (10)	−0.0034 (8)	0.0077 (8)	−0.0014 (9)
C13	0.0591 (13)	0.0512 (12)	0.0338 (10)	0.0060 (10)	0.0136 (9)	0.0021 (9)
C14	0.0555 (12)	0.0523 (12)	0.0405 (11)	0.0069 (9)	0.0143 (9)	−0.0007 (9)
C15	0.0530 (12)	0.0501 (12)	0.0417 (11)	0.0074 (9)	0.0157 (9)	0.0048 (9)
C16	0.0606 (13)	0.0563 (13)	0.0370 (11)	0.0024 (10)	0.0169 (9)	0.0022 (10)
C17	0.0556 (12)	0.0484 (12)	0.0424 (11)	0.0014 (9)	0.0052 (9)	0.0074 (9)
C18	0.0687 (14)	0.0573 (13)	0.0498 (12)	−0.0091 (10)	0.0000 (10)	−0.0008 (10)
C19	0.0644 (15)	0.0843 (16)	0.0499 (12)	−0.0054 (11)	−0.0041 (10)	0.0061 (12)
C20	0.0703 (15)	0.0679 (15)	0.0481 (12)	−0.0004 (11)	0.0033 (10)	0.0008 (11)
C21	0.0822 (16)	0.0874 (17)	0.0520 (13)	−0.0048 (12)	−0.0011 (11)	0.0134 (12)
C22	0.1049 (19)	0.0948 (19)	0.0611 (15)	−0.0152 (14)	0.0048 (13)	0.0105 (13)

### Geometric parameters (Å, °)

**Table:**

F—C1	1.3629 (19)	C13—C14	1.352 (2)
N1—C9	1.148 (2)	C13—H13A	0.9300
N2—C10	1.153 (2)	C14—H14A	0.9300
N3—C14	1.3493 (19)	C15—C16	1.349 (2)
N3—C16	1.353 (2)	C15—H15A	0.9300
N3—C17	1.4793 (19)	C16—H16A	0.9300
C1—C2	1.356 (2)	C17—C18	1.506 (2)
C1—C6	1.364 (2)	C17—H17A	0.9700
C2—C3	1.383 (2)	C17—H17B	0.9700
C2—H2A	0.9300	C18—C19	1.526 (2)
C3—C4	1.391 (2)	C18—H18A	0.9700
C3—H3A	0.9300	C18—H18B	0.9700
C4—C5	1.386 (2)	C19—C20	1.510 (2)
C4—C7	1.487 (2)	C19—H19A	0.9700
C5—C6	1.386 (2)	C19—H19B	0.9700
C5—H5A	0.9300	C20—C21	1.519 (2)
C6—H6A	0.9300	C20—H20A	0.9700
C7—C11	1.384 (2)	C20—H20B	0.9700
C7—C8	1.407 (2)	C21—C22	1.508 (2)
C8—C9	1.411 (2)	C21—H21A	0.9700
C8—C10	1.419 (2)	C21—H21B	0.9700
C11—C12	1.417 (2)	C22—H22A	0.9600
C11—H11A	0.9300	C22—H22B	0.9600
C12—C13	1.411 (2)	C22—H22C	0.9600
C12—C15	1.413 (2)		
			
C14—N3—C16	118.26 (14)	C16—C15—H15A	118.8
C14—N3—C17	121.48 (15)	C12—C15—H15A	118.8
C16—N3—C17	120.24 (14)	C15—C16—N3	121.49 (16)
C2—C1—F	118.64 (18)	C15—C16—H16A	119.3
C2—C1—C6	123.51 (17)	N3—C16—H16A	119.3
F—C1—C6	117.85 (18)	N3—C17—C18	111.79 (14)
C1—C2—C3	118.38 (17)	N3—C17—H17A	109.3
C1—C2—H2A	120.8	C18—C17—H17A	109.3
C3—C2—H2A	120.8	N3—C17—H17B	109.3
C2—C3—C4	120.85 (17)	C18—C17—H17B	109.3
C2—C3—H3A	119.6	H17A—C17—H17B	107.9
C4—C3—H3A	119.6	C17—C18—C19	112.70 (15)
C5—C4—C3	118.16 (15)	C17—C18—H18A	109.1
C5—C4—C7	121.33 (15)	C19—C18—H18A	109.1
C3—C4—C7	120.47 (15)	C17—C18—H18B	109.1
C6—C5—C4	121.53 (16)	C19—C18—H18B	109.1
C6—C5—H5A	119.2	H18A—C18—H18B	107.8
C4—C5—H5A	119.2	C20—C19—C18	115.26 (16)
C1—C6—C5	117.55 (17)	C20—C19—H19A	108.5
C1—C6—H6A	121.2	C18—C19—H19A	108.5
C5—C6—H6A	121.2	C20—C19—H19B	108.5
C11—C7—C8	120.62 (15)	C18—C19—H19B	108.5
C11—C7—C4	122.68 (14)	H19A—C19—H19B	107.5
C8—C7—C4	116.66 (14)	C19—C20—C21	112.76 (16)
C7—C8—C9	123.00 (16)	C19—C20—H20A	109.0
C7—C8—C10	120.83 (15)	C21—C20—H20A	109.0
C9—C8—C10	116.15 (15)	C19—C20—H20B	109.0
N1—C9—C8	178.3 (2)	C21—C20—H20B	109.0
N2—C10—C8	177.3 (2)	H20A—C20—H20B	107.8
C7—C11—C12	130.83 (16)	C22—C21—C20	112.99 (17)
C7—C11—H11A	114.6	C22—C21—H21A	109.0
C12—C11—H11A	114.6	C20—C21—H21A	109.0
C13—C12—C15	114.01 (15)	C22—C21—H21B	109.0
C13—C12—C11	127.40 (15)	C20—C21—H21B	109.0
C15—C12—C11	118.49 (16)	H21A—C21—H21B	107.8
C14—C13—C12	121.53 (16)	C21—C22—H22A	109.5
C14—C13—H13A	119.2	C21—C22—H22B	109.5
C12—C13—H13A	119.2	H22A—C22—H22B	109.5
N3—C14—C13	122.33 (17)	C21—C22—H22C	109.5
N3—C14—H14A	118.8	H22A—C22—H22C	109.5
C13—C14—H14A	118.8	H22B—C22—H22C	109.5
C16—C15—C12	122.35 (17)		
			
F—C1—C2—C3	179.02 (15)	C4—C7—C11—C12	14.2 (3)
C6—C1—C2—C3	−0.9 (3)	C7—C11—C12—C13	11.4 (3)
C1—C2—C3—C4	0.6 (3)	C7—C11—C12—C15	−172.34 (16)
C2—C3—C4—C5	0.6 (2)	C15—C12—C13—C14	1.1 (2)
C2—C3—C4—C7	178.29 (15)	C11—C12—C13—C14	177.44 (16)
C3—C4—C5—C6	−1.6 (2)	C16—N3—C14—C13	−0.1 (3)
C7—C4—C5—C6	−179.27 (15)	C17—N3—C14—C13	178.42 (15)
C2—C1—C6—C5	−0.1 (3)	C12—C13—C14—N3	−0.2 (3)
F—C1—C6—C5	−179.97 (15)	C13—C12—C15—C16	−1.7 (2)
C4—C5—C6—C1	1.4 (3)	C11—C12—C15—C16	−178.44 (16)
C5—C4—C7—C11	−125.40 (18)	C12—C15—C16—N3	1.6 (3)
C3—C4—C7—C11	57.0 (2)	C14—N3—C16—C15	−0.6 (3)
C5—C4—C7—C8	57.0 (2)	C17—N3—C16—C15	−179.09 (15)
C3—C4—C7—C8	−120.63 (17)	C14—N3—C17—C18	−99.45 (19)
C11—C7—C8—C9	−170.88 (16)	C16—N3—C17—C18	79.0 (2)
C4—C7—C8—C9	6.8 (2)	N3—C17—C18—C19	173.21 (15)
C11—C7—C8—C10	7.7 (2)	C17—C18—C19—C20	63.6 (2)
C4—C7—C8—C10	−174.63 (15)	C18—C19—C20—C21	173.53 (17)
C8—C7—C11—C12	−168.25 (17)	C19—C20—C21—C22	−175.80 (17)

### Hydrogen-bond geometry (Å, °)

**Table:**

*D*—H···*A*	*D*—H	H···*A*	*D*···*A*	*D*—H···*A*
C3—H3A···N2^i^	0.93	2.61	3.535 (3)	175
C16—H16A···N1^ii^	0.93	2.51	3.354 (3)	151

Symmetry codes: (i) −*x*+1, −*y*, −*z*; (ii) *x*, −*y*+1/2, *z*−1/2.
